# Elicitation, an Effective Strategy for the Biotechnological Production of Bioactive High-Added Value Compounds in Plant Cell Factories

**DOI:** 10.3390/molecules21020182

**Published:** 2016-02-03

**Authors:** Karla Ramirez-Estrada, Heriberto Vidal-Limon, Diego Hidalgo, Elisabeth Moyano, Marta Golenioswki, Rosa M. Cusidó, Javier Palazon

**Affiliations:** 1 Laboratori de Fisiologia Vegetal, Facultat de Farmacia, Universitat de Barcelona, Av. Joan XXIII sn, Barcelona 08028, Spain; kems3estrada@gmail.com (K.R.-E.); bioraf@hotmail.com (H.V.-L.); diegoahmz@hotmail.com (D.H.); elisabeth.moyano@upf.edu (E.M.); rcusido@ub.edu (R.M.C.); 2 Argentina National Rechearch Council (Conicet), Ministry of Science and Technology, Arenales 230. B. Junior, Córdoba X5000APP, Argentina; mgoleniowski@gmail.com

**Keywords:** plant cell factories, plant secondary metabolism, secondary compound production, elicitors

## Abstract

Plant *in vitro* cultures represent an attractive and cost-effective alternative to classical approaches to plant secondary metabolite (PSM) production (the “Plant Cell Factory” concept). Among other advantages, they constitute the only sustainable and eco-friendly system to obtain complex chemical structures biosynthesized by rare or endangered plant species that resist domestication. For successful results, the biotechnological production of PSM requires an optimized system, for which elicitation has proved one of the most effective strategies. In plant cell cultures, an elicitor can be defined as a compound introduced in small concentrations to a living system to promote the biosynthesis of the target metabolite. Traditionally, elicitors have been classified in two types, abiotic or biotic, according to their chemical nature and exogenous or endogenous origin, and notably include yeast extract, methyl jasmonate, salicylic acid, vanadyl sulphate and chitosan. In this review, we summarize the enhancing effects of elicitors on the production of high-added value plant compounds such as taxanes, ginsenosides, aryltetralin lignans and other types of polyphenols, focusing particularly on the use of a new generation of elicitors such as coronatine and cyclodextrins.

## 1. Introduction

Plants produce a huge amount of compounds that are not strictly necessary for growth and development, but which play a crucial role in defense and adaptation to the environment. These secondary metabolites, which include terpenes, steroids, phenolics and alkaloids, exhibit a wide range of biological activities and have immense potential application in the chemical-pharmaceutical industries as pharmaceuticals, agrochemicals, flavors, fragrances, colors, biopesticides, and food additives [1,2].

For ecological, political or geographical reasons, the plant raw materials that are the sources of some of these valuable compounds are becoming increasingly short in supply. The production of a metabolite is often restricted to a concrete species or genus and might be activated only during a particular growth or developmental stage, or under specific conditions related to the season, stress or nutrient availability. If difficult to cultivate, plants are collected in the field, with a consequent risk of extinction. Moreover, certain species grow very slowly, for example, *Panax ginseng* roots need about six years before they are ready for harvesting [3], while *Taxus* trees reach a peak production of taxol only after 60 years of growth [4]. Consequently, even after considerable long-term planning, market demands for target compounds can be difficult to meet. Further, the extraction of fine chemicals from plants can be very challenging and expensive, giving poor yields. For all these reasons, in the last decades considerable effort has been invested in the biotechnological production of metabolites by means of plant cell and organ cultures.

Plant cell factories constitute the most promising approach for a sustainable production of plant secondary metabolites of commercial interest [5,6], offering a continuous supply by means of large-scale culture. They have several advantages over the cultivation of medicinal and aromatic plants in the field [6]: (a) the desired product can be harvested anywhere in the world with strict control of production and quality; (b) independence from geographical or environmental fluctuations; (c) uncontaminated plant material is guaranteed, since plant cells are free of microorganisms, herbicides, pesticides and fungicides; (d) endangered plant species can be conserved for future generations; (e) growth cycles are of weeks rather than years as in the intact plant.

Yet despite all these advantages, and the extensive effort invested in developing plant cell cultures as PSM production systems, commercially successful plant cell factories are still rare, which is at least partly due to a lack of knowledge of plant secondary metabolism and its *in vitro* control. The few industrially viable processes established to date produce pure compounds such as shikonin [7], taxol [8,9] and berberine [10], or biomass, as in the case of ginseng roots [11]. An essential challenge for such a biotechnological system is to maintain costs below that of large-scale cultivation of plants. Given the complexity of *in vitro* cultivation techniques, together with the cost of sterilization and culture in bioreactors, new technologies are applied almost exclusively for the production of value-added compounds.

The profitability of industrial PSM production depends largely on system productivity. In general, when cell or organ cultures are first established, yields are relatively low. Most attempts to increase biotechnological production have been based on an empirical approach, using a range of methodologies to enhance metabolite biosynthesis and accumulation. An alternative approach is to gain an understanding of the metabolic pathways involved.

A protocol for the large-scale production of valuable secondary compounds first of all requires the establishment of callus biomass from selected highly productive plant genotypes. Calli, which are formed mainly by stem cells, have an unlimited growth capacity and can synthesize the same compounds as the original plant. Cell suspensions derived from the calli are first maintained on a small scale and then at bioreactor level. In all these steps, the culture conditions and environmental and physical factors (such as light, pH, temperature, shaking speed, *etc.*) need to be optimized to achieve a high production by assaying different culture media, hormonal combinations and carbon sources [12].

In the exponential growth phase of plant cell cultures, many metabolites are produced only at low levels, or not all, since their primary metabolite precursors are required for biomass formation. There is evidence that the induction of secondary metabolite production from primary compounds is more effective in the stationary growth phase. For this reason, a good strategy for a plant cell factory is to establish a two-stage culture, in which the cells are first maintained in an optimal medium for biomass formation and are then transferred to an optimal production medium that stimulates the synthesis of secondary compounds. This system has the advantage of allowing elicitors and biosynthetic precursors to be added at the time of maximum yield, that is, in the second phase of the culture [13,14].

One of the most effective strategies for enhancing the biotechnological production of secondary compounds is elicitation. Although a cell culture can be elicited by physical factors, the addition of biotic or abiotic elicitors to the culture medium is the main methodology used in biotechnological cell cultures. Since it is impossible to consider the great variety of elicitors assayed in plant cell cultures in their entirety, in this review we have focused mainly on the action of the most commonly used and effective biotic elicitors for the biosynthesis and accumulation of secondary compounds of great interest for chemical-pharmaceutical industries.

## 2. Elicitation

Elicitation is one the most effective techniques currently used for improving the biotechnological production of secondary metabolites. Elicitors are compounds that stimulate any type of plant defense, promoting secondary metabolism to protect the cell and the whole plant [15,16,17]. According to their origin, elicitors can be divided into different types: (a) biotic and (b) abiotic. Abiotic elicitors can be considered as substances of non-biological origin, being predominantly inorganic compounds such as salts or physical factors [18,19]. Inorganic chemicals like salts or metal ions have been used to increase the production of bioactive compounds by their modification of plant secondary metabolism. Salts (including AgNO_3_, AlCl_3_, CaCl_2_, CdCl_2_, CoCl_2_, CuCl_2_, HgCl_2_, KCl, MgSO_4_, NiSO_4_, VOSO_4_ and Zn ions) can elicit PSM production in a variety of plant species in culture systems such as cell suspensions, hairy roots and adventitious roots [20]. In this review, however, we will be focusing on the more extensively explored biotic elicitors.

The majority of biotic elicitors are recognized by specific receptors bound to the cell membrane. This stimulus is then transferred to the cell by a signal transduction system, producing changes that ultimately lead to the formation of phytoalexins [17]. The response of the plant is determined by several factors, principally its genetic characteristics and physiological state. In general, plant resistance to disease is controlled by plant resistance (R) and pathogen avirulence (Avr) genes [21]. However, while specific Avr products trigger defense responses in cultivars with matching R genes, the action of general elicitors can activate defenses in cultivars of more than one species [22].

According to the new concept of plant innate immunity, defense responses are triggered when plant cells recognize conserved microbe-associated molecular patterns (MAMPs), a new term for general and exogenous elicitors. Alternatively, a pathogen invasion can prompt the release of plant endogenous molecules/endogenous elicitors, termed as danger-associated molecular patterns. A second level of perception involves the recognition of pathogen-secreted effectors, formerly known as specific elicitors, which belong to different families, including proteins, glycans and lipids [23].

## 3. General Mechanism of Action of Elicitors

The response of plants to elicitor-induced stress usually begins at the cell plasma membrane. Although there are several receptors in the cytosol, associated with both the nucleus and cytosolic membrane, in this review we comment briefly only on the plasmalemma membrane-associated elicitors, since in cell cultures these are among the most studied. Considerable effort has been invested to isolate elicitor signal molecules and identify the corresponding receptors. As elicitors can induce reactions in different species, it would seem that plants have common receptors for their perception. Several elicitor-binding sites have been identified in cell plasma membranes for a range of elicitors of different chemical structures. R and avr gene products play a key role in this step [22].

**Figure 1:**
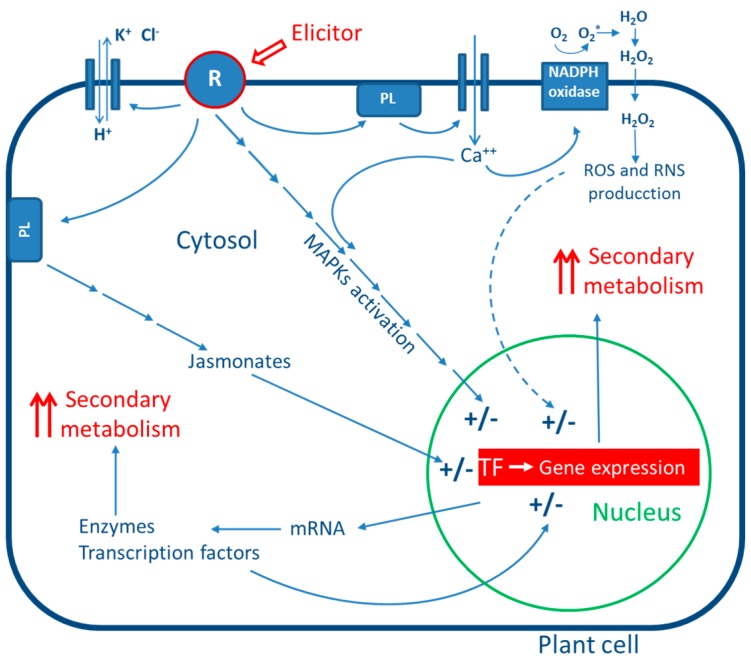
Schematic representation of the possible responses of cells to elicitation. R: receptor; PL: phospholipase; MAPKs: mitogen activated protein kinases; ROS: reactive oxygen species; RNS: reactive nitrogen species; TF: transcription factors.

The transduction of the elicitor signal perceived by the receptors entails the action of second messengers, which further amplify the signal for other downstream reactions [24]. The sequentially occurring events in elicitor-induced defense responses can be summarized as follows (Figure 1): elicitor perception by the receptor; reversible phosphorylation and dephosphorylation of plasma membrane proteins and cytosolic proteins; cytosolic [Ca^2+^] enhancement; Cl^−^ and K^+^ efflux/H^+^ influx: extracellular alkalinization and cytoplasmic acidification; mitogen-activated protein kinase (MAPK) activation; NADPH oxidase activation and production of reactive oxygen and nitrogen species (ROS and RNS) [16]; early defense gene expression; jasmonate production; late defense response gene expression, and secondary metabolite accumulation [25,26,27] (Figure 1). The systemic responses used by plants to prevent attacks lead to the production of antimicrobial compounds such as phytoalexins and pathogenesis-related (PR) proteins, which together play a key role in pathogen rejection [25]. Elicitor perception can also increase the level of plant resistance against future pathogen attack.

Thus, elicitor signal transduction is a multiple component network that establishes an efficient defense by various sequential reactions. These multiple components consist of parallel or cross-linking signaling pathways leading to different target responses. An elicitor-signaling pathway may vary in the perception of different elicitor signals or target defense responses. Hypertensive responses can also take place, characterized by rapid cell death in the immediate vicinity of the point of exposure to the pathogen [27], as well as the formation of structural defensive barriers, such as lignin deposition to reinforce cell walls.

Considerable research is being devoted to the elucidation of the mechanism of action of the main elicitors currently applied in plant biotechnology. Although the receptors, secondary messengers, transduction pathways and responsive genes have been determined in some cases, in general the data is very incomplete. Moreover, the variability of these mechanisms of action entails a wide range of metabolic responses. Due to this complexity, most of the studies about the enhancing effect of elicitation on secondary compound production in plant cell cultures have been empirical, without exploring the cellular response at a molecular level.

Although the aforementioned signaling components are reported to be involved in elicitor signaling pathways towards PSM production, not all of them will be used in a particular plant or for the production of a specific metabolite. Studies are increasingly revealing that a defensive cellular process is usually regulated by two or more signaling pathways working in collaboration. As mentioned above, cross-talk among multiple signaling [16] pathways is an important mechanism in plant signal transduction networks, enabling plants to regulate different sets of genes temporally and spatially in a range of situations against many types of stress.

## 4. Biotic Elicitors

The term elicitor was originally used for molecules capable of inducing the production of phytoalexins, but it now commonly refers to compounds stimulating any type of plant defense [28,29]. This broader definition includes substances of pathogenic origin (exogenous elicitors) and compounds produced by plants after the action of the pathogen (endogenous elicitors) [28,30,31].

The application of biotic elicitors, that is, those of biological origin, in plant cell cultures constitutes an excellent system to enhance the production of secondary compounds with phytoalexinic properties, as well as to obtain more insight into the regulation of their biosynthetic pathways.

Exogenous biotic elicitors include compounds released by microorganisms and other pathogens, or formed by the action of plant enzymes on microbial cell walls, e.g., microbial enzymes, fungal and bacterial lysates, yeast extracts, and polysaccharides from microorganism cell walls (e.g., chitin and glucanes). Those of endogenous origin include polysaccharides arising from pathogen degradation of the plant cell wall, intracellular proteins, and small molecules synthesized by the plant cell in response to different types of stress or pathogen attack, including plant hormones such as methyl jasmonate or salicylic acid.

Presented here is a comprehensive overview of the principal biotic elicitors that have been tested, separately and in combination, in a wide range of plant cell cultures to improve PSM production. They have been divided into the following groups: (a) plant hormones; (b) microorganism-derived elicitors; (c) plant cell wall fragments; (d) others (Table 1). Attention has been given primarily to studies of the last two decades on the biotechnological production of high added value PSM, such as lignans, ginsenosides and taxanes.

### 4.1. Plant Hormones

#### 4.1.1. Methyl Jasmonate

Jasmonates (JAs) are plant-specific signaling molecules that activate several important physiological and developmental processes [32]. The biosynthesis of these hormones, which is induced by pathogen attack and wounding, triggers defense responses, both locally and systemically. JAs, particularly methyl jasmonate (MeJa), have been reported to play an important role in signal transduction processes that regulate defense genes in plants [33]. When exogenously applied to plant cell cultures of a variety of species, MeJa (100–200 μM) positively stimulates the workflow of secondary biosynthetic pathways, leading to an increased production of diverse PSM, including terpenoids, flavonoids, alkaloids and phenylpropanoids [34,35,36]. An effective application of MeJa requires empirical studies to find the optimum dose and time of supplementation.

A PSM of pharmaceutical interest, whose production is clearly enhanced by the addition of MeJa to the culture medium, is the aryltetralignan podophyllotoxin. The principal source of podophyllotoxin is the *Podophyllum* genus of the Berberidaceae family, which includes six species of perennial herbaceous plants native to eastern Asia (five species) and eastern North America (one species, *P. peltatum*). It is also synthezised in plants of other genera, including *Jeffersonia*, *Diphylleia*, *Dysosma*, *Catharanthus*, *Polygala*, *Anthriscus* and *Linum* [37].

The pharmacological properties reported for podophyllotoxin and its analogs include cytotoxicity, and antiviral and antitumoral activities [38,39]. A *Linum* species endemic to the Balkans has been traditionally used to treat cancer, and the genus *Linum* has become one of the most studied alternatives for podophyllotoxin production. Van Fürden *et al.* [40] reported an increment in podophyllotoxin and 6-methoxypodophyllotoxin accumulation in cell suspension cultures of *Linum album* cell line 2-5aH after elicitation with MeJa. Exogenous addition of MeJa to *L. tauricum* hairy root cultures increased the 4′-dimethyl-6-methoxypodo-phylotoxin and 6-methoxypodophyllotoxin yield 1.2-fold [41].

Another group of PSM whose production has been improved in biotechnological systems are the ginsenosides, triterpene saponins that are the primary pharmacologically active ingredients in ginseng [42]. The roots of *Panax ginseng*, a perennial herb of the Araliceae family, are well-known for their use as a health food and in traditional medicine. Among the various pharmacological effects attributed to ginseng are enhanced immunity, stamina, general health and resistance to stress [1,2,3]. However, despite extensive studies on the optimization of growth and yield, production of this valuable pharmaceutical remains low. Improvements in the accumulation of ginsenosides in *P. ginseng* hairy root cultures have been achieved using MeJa elicitation [43,44]. A wide range of concentrations have been tested, alone and in combination with other compounds, resulting in a more than 10-fold increment in production [45,46,47,48,49,50]. Notably, Thanh *et al.* [51] achieved an increase in ginseng yield of up to 28-fold in MeJa-elicited cell suspension cultures.

Biotechnological systems with a strong response to JA application are cell cultures of *Taxus* spp., currently used for the production of paclitaxel and related taxanes. Paclitaxel, a diterpene alkaloid that accumulates in the inner bark of *Taxus* spp., has shown high effectivity against several cancer processes, including ovary, breast and prostate, as well as other illnesses related with microtubular exchange [1,2]. Treatment of *Taxus* cell cultures with MeJa is one of the most effective strategies for boosting taxane production [52], although it can limit biomass formation. In fact, *Taxus* cell cultures were one of the first *in vitro* systems to give positive results after the addition of MeJa to the culture medium [53]. Considerable research efforts have been dedicated to optimizing conditions for the best cellular response to MeJa elicitation. Cell immobilization and precursor addition [54], the combination of MeJa with abiotic elicitors like the novel cyclodextrins (CD) [55,56], or the use of transformed hairy roots [57] are some of the strategies that have given very promising results. At present, several pharmaceutical companies (Phyton, Cell Therapeutics, Abraxis, Corean Samyang Genex, *etc.*) commercialize paclitaxel obtained in elicited plant cell cultures at bioreactor level. [58].

Other bioactive compounds whose yield is enhanced by MeJa include the tropane alkaloids scopolamine and hyoscyamine in transgenic hairy roots of *Hyoscyamus niger* [59], and stilbenes and t-resveratrol in cell cultures of *Vitis vinifera* [60]. It has also been applied to increase the accumulation of the phenylpropanoid rosmarinic acid in cell suspension cultures of *Mentha x piperita* and *Lavandula vera* [61,62]. Elicitation studies have shown that MeJa can improve the production of peruvoside, a cardiac glycoside, in cell suspension cultures of *Thevetia peruviana* [63] as well as that of catharanthine, one of the two precursors of the anticancer compound vinblastine, in *Catharantus roseus* hairy roots [64]. Studies with MeJa-elicited *Centella asiatica* hairy roots and cell suspensions obtained a highly increased production of centellosides [65,66]. Likewise, MeJa treatment of *Artemisia annua* cell cultures stimulated the synthesis of artemisinin [67]. These are only a few examples of the successful use of MeJa elicitation in plant cells to improve PSM productivity.

#### 4.1.2. Salicylic Acid

Salicylic acid (SA), a small molecule with a vital role in plant defense regulatory systems, is known to induce systemic acquired resistance (SAR) to many pathogens [68,69,70]. During the plant-pathogen interaction, a rapid SA accumulation in the infection site triggers a hypersensitive response. The signal then spreads to other parts of the plant to induce a wide range of defense responses [36], including the production of PSM, which is why SA is widely applied as a secondary metabolism elicitor [19,71]. Nevertheless, SA is not a global elicitor, and induces only certain classes of PSM [36].

Numerous studies on the *Taxus* genus have applied SA elicitation to enhance diterpene alkaloid production. Wang *et al.* [72] treated *T. chinensis* cell suspension cultures with an optimal concentration of 20 mg/L SA to induce paclitaxel production. Improved yields of this taxane were also achieved in a two-stage cell suspension culture of *T. baccata* using two different concentrations of SA [73]. SA has also been tested in combination with a magnetic field to improve the paclitaxel yield in a *T. chinensis* cell suspension [74]. The production of this anticancer compound in *Corylus avellana* cell cultures was also successfully enhanced by SA [75].

SA has been reported as an effective elicitor of other types of secondary metabolites in other plant genera. In SA-treated *Linum album* cell cultures, podophyllotoxin production was 3-fold higher than in control cultures after 3 days [76]. Ginsenoside production was improved by SA elicitation in *P. ginseng* adventitious roots [77,78,79] and hairy roots, in the latter even at low doses [80]. SA addition induced the accumulation of sesquiterpenes, including bilobalide and ginkgolide A and B, in cell suspension cultures of *Ginkgo biloba* [81]. After SA elicitation of *Hypericum* spp. cell suspension and hairy root cultures, the production of xanthonescadensin G and paxanthone increased about 2-fold [82]. Stilbene production in cell suspension cultures of *V. vinifera* was positively affected by SA, alone or in combination with other treatments [83]. Adventitious roots of *Withania somnifera* showed an increment in production of the anti-inflammatory withanolides after SA treatment [84]. The yields of the anti-cancer compound dicentrine in *Stephania venosa* [85] and naphtodianthrones in *Hypericum perforatum* [86] cell cultures were improved with SA elicitation.

#### 4.1.3. SA Derivatives and Analogs

SA derivatives and analogs, such as 2,6-dichloroisonicotinic acid (INA) and BTH derivatives [87,88,89], have also been used as chemical inducers of SAR to protect plants from infections from fungal, bacterial, or viral pathogens [90].

Benzothiadiazole (BTH) derivatives protect plants from diseases by activating the SA signaling pathway. Acibenzolar-*S*-methyl (ASM), a compound of this group developed for plant protection, does not have antimicrobial properties, but instead increases resistance to diseases by triggering SAR in several crop species, including bean, cauliflower, cucumber, tobacco, apple and pear [91,92]. Treatment of *Solanum malacoxylon* cell cultures with 50 μM benzothiadiazole resulted in increased cellular levels of β-sitosterol, campesterol, stigmasterol, sterol and vitamin D (3) [93].

The elicitation of parsley (*Petroselinum crispum*) cell suspensions with 2,6-dichloroisonicotinic, 4- or 5-chlorosalicylic and 3,5-dichlorosalicylic acid induced the incorporation of phenylpropanoid derivatives into the cell wall and, indirectly, the secretion of soluble coumarin derivatives [94]. The preincubation of the cultures with these chemicals greatly increased the subsequent induction of coumarin derivatives by a fungal elicitor, especially at low elicitor concentrations and in cell batches exhibiting a low response due to unknown variations in growth conditions. The effect is apparently the result of increased cell sensitivity towards the elicitor, which causes enhanced transcription of genes encoding enzymes involved in coumarin synthesis.

#### 4.1.4. Brassinosteroids

Brassinosteroids (BRs) are essential for plant growth and development. In general, such hormones act via a soluble receptor/ligand complex that binds to nuclear sites to regulate the expression of specific genes. Due to structural similarity, it is proposed that they act by a mechanism comparable to that of animal steroid hormones [95]. It has long been established that BRs elicit a variety of effects on the growth of higher plants [96,97]. In cell suspension cultures of *Ornithopus sativus*, BRs produced acyl-conjugated metabolites [98]. Although not recently, several studies have also been carried out on the elicitor effect of BRs in cell cultures of *Raphanus sativus* and *Secale cereale* [99].

### 4.2. Microorganism-Derived Elicitors

Included among biotic elicitors are those derived from the pathogen itself. In the initial studies on the enhancement of PSM production in plants, elicitation was performed using biological and pathogen mixtures [19], since it was known that many secondary metabolic pathways are induced by infections with microorganisms. Researchers continue to use fungal, yeast and bacterial extracts due to the good results obtained, often in complex biological preparations without knowledge of the molecular structure of the ingredients. Among these, bacterial extracts and yeast and fungal extracts are included in this review.

#### 4.2.1. Bacterial Extracts

Bacterial extracts consist of biological mixtures prepared from autoclaved and centrifuged microorganism cultures, without identification of the active compounds. They have been successfully applied to enhance the production of ginsenosides in *P. ginseng* hairy roots [80]. *Staphylococcus aureus* extracts enhanced bilobalide and ginkgolide biosynthesis in *G. biloba* cell suspension cultures [100] and *Beta vulgaris* hairy roots increased betalain accumulation after treatment with extracts of whole microbial cultures [101]. Co-culture is another strategy to improve PSM production: for example, tanshinone production was stimulated in *Salvia miltiorrhiza* hairy roots co-cultured with *Bacillus cereus* [102].

#### 4.2.2. Yeast Extract and Fungal Elicitors

Despite limited knowledge of the composition and mechanism of action of yeast and fungal elicitors, autoclaved solutions of yeast culture, fungi mycelia or spores providing cell wall fragments are widely used as elicitors to enhance PSM production, mainly in plant cell or hairy root cultures. An extract of the yeast *Aureobasidium pullulans* stimulated the accumulation of stilbene phytoalexins in *V. vinifera* calli [103]. Treatment of *P. ginseng* cell suspension and hairy root cultures with a yeast preparation enhanced the content of saponins and other unknown compounds [28,80]. Yeast polysaccharides stimulated the production of flavonoids in hairy roots derived from *Fagopyrum tataricum* [104].

Among many studies reporting positive effects of fungal elicitation on PSM production, Gadzovska Simic *et al.* [105] observed a significant increase in naphtodianthrones, total phenolics, flavonoids and anthocyanins in treated *H. perforatum* cell suspensions. The yield of forskolin increased up to 6-fold in cell suspension cultures of *Coleus forskohlii* after elicitation with several fungal elicitors [106]. Fungal preparations from *Aspergillus niger* and *Penicillium notatum* extract increased the synthesis of psoralen in 16-day old cell cultures of *Psoralea corylifolia* [107]. Terpenoid and polyphenol biosynthesis was also stimulated by an endophytic fungal elicitor in *Euphorbia pekinensis* cell suspensions [108]. Mendhulkar and Vakil [109] reported a significant increase of flavonoid accumulation in suspension cultures of *Andrographis paniculata* after elicitation with an *Aspergillus niger* extract. Several fungal extracts have also been successfully used to improve lignan production in cell suspensions of *Linum* species [110,111]. Bahabadi *et al.* [112,113] tested the elicitation effect of five different fungal extracts, which proved more efficient than pure elicitors like chitosan and chitin.

Another commonly used elicitor is commercial yeast extract produced by the digestion of yeast by exogeneous or endogenous enzymes or in acid conditions. Examples of its successful application to improve PSM production in cell suspension cultures include podophyllotoxin in *Linum album* [29], stilbenes in *Cayratia trifolia* [114] and silymarin in *Silybum marianum* [30]. In hairy roots treated with yeast extract, improvements have been obtained for artemisinin in *A. annua* [115] and rosmarinic acid in *S. miltiorrhiza* [116].

Cell cultures of several *Taxus* species have been treated with various fungal extracts, alone or in combination with other elicitors, to boost yields of paclitaxel and other taxanes [117,118,119]. *P. ginseng* cell suspension cultures have also been treated with different strains and kinds of fungal elicitors, obtaining significant increments [120]; hairy root cultures also responded positively [121].

An interesting strategy is the co-cultivation of cell suspensions with fungal cultures; for example, the culture of *L. album* with mycorrhiza-like fungi [122] or *T. chinensis* with its endophytic fungi [123] in a co-bioreactor enhanced the production of podophyllotoxin and 6-methoxypodophyllotoxin and paclitaxel, respectively. When a root endophytic fungus was added to *L. album* hairy root cultures, lignin production improved significantly [124].

#### 4.2.3. Bacteria and Fungi-Derived Peptides and Proteins

Cerato-platanins (CP) are small, cysteine-rich fungal-secreted proteins involved in the various stages of the host-fungus interaction process, acting as phytotoxins, elicitors, and allergens [125]. Due to these intriguing properties, they have potential application as elicitors in plant cell culture.

Harpins are glycine-rich and heat-stable proteins secreted from certain plant-pathogenic bacteria, and act mainly in the extracellular space of plant tissues, not inside the plant cells like bacterial effector proteins. Some harpins have virulence activity, probably because of their involvement in the translocation of effector proteins into plant cytoplasm [126]. Several reports have shown defense-related responses to these compounds, such as lignification, phytoalexin production, lipid peroxidation, and oxidative cross-linking of cell-wall structural proteins [127,128]. Recently, harpins have been described as the elicitor responsible for the HR caused by plant-pathogenic bacteria [126].

The first described use of coronatine as an elicitor was by Weiler *et al.* [129], who found that this phytoxin, produced by several patovars of *Pseudomonas syringae*, was able to induce the accumulation of defense-related PSM in several cell cultures. Due to its chemical structure, coronatine acts as a molecular mimic of the isoleucine-conjugated form of jasmonic acid (JA–Ile) [130], while being more stable [131], and consequently has a similar mechanism of action to the elicitor MeJa. Interestingly, cultures are generally more productive when treated with coronatine than with higher concentrations of MeJa in the same culture conditions.

Tamogami and Kodama [132] reported an induced accumulation of some flavonoid phytoalexins when rice leaves were treated with different concentrations of coronatine (0.05–0.4 mM). The effect of coronatine on flavonoid production was greater than that of JA or 12-oxo-phytodienoic acid (PDA) (all at 0.5 mM). Haider *et al.* [133] showed the positive action of coronatine and some structural analogues on benzo[*c*]phenanthridine alkaloid production in *Eschscholzia californica* cell cultures, although in these studies coronatine had a lower elicitor effect than MeJa and some analogues. The accumulation of glyceollins, soybean phytoalexins (*Glycine max* L.), in soybean cell suspension cultures was studied after the addition of several elicitors related with the JA biosynthetic pathway. JA and MeJa showed weak phytoalexin-inducing activity compared to an early jasmonate biosynthetic precursor, the PDA, or the bacterial phytotoxin coronatine and certain 6-substituted indanoyl-l-isoleucine methyl esters, which were all highly active [134,135].

Coronatine usually activates PSM production in cell cultures at concentrations lower than MeJa. In *Corylus avellana* cell suspensions, very low amounts of coronatine stimulated taxane production up to 27-fold [136]. In *T. media* cell cultures, coronatine at 1 μM had a remarkable effect on the taxane production, which increased almost 10- and 4-fold compared with the same cell line cultured in control conditions or supplemented with 100 μM MeJa, respectively [137]. In a comparison of *T. globosa* and *T. media* cell line behavior, the stimulative effect of coronatine was found to be enhanced by co-treatment with an abiotic elicitor. [131,138]. In *L. album* cell suspension cultures, coronatine produced a high increase in podophyllotoxin and 6-methoxypodophyllotoxin [139]. The effect of elicitation with two synthetic derivatives of coronatine was studied in cell suspensions of *Linum nodiflorum*, obtaining an increment of more than 10-fold in 6-methoxypodophyllotoxin yield compared to the control [140].

### 4.3. Cell Wall-Derived Elicitors

#### 4.3.1. Chitosan and Chitin

Purified components from complex crude fungus or yeast extracts and their derivatives have also been used as elicitors, such as the carbohydrates chitosan and chitin. Chitin is a long-chain polysaccharide of β-(1→4)-*N*-acetyl-d-glucosamine units synthesized by a huge number of living organisms, including fungi and yeast, in which it is a characteristic cell wall component. Chitosan, the most important derivative of chitin, is obtained by partial deacetylation of solid chitin under alkaline conditions or by enzymatic hydrolysis with chitin deacetylase [141].

In *L. album* cell cultures podophyllotoxin production improved after elicitation with both chitin and chitosan [112], and increased expression of the genes involved in podophyllotoxin biosynthesis was observed [142]. *T. chinensis* cell suspensions supplemented separately with chitosan and chitin improved paclitaxel production [143,144], while MeJa elicitation in *T. canadiensis* cell cultures was enhanced by these polysaccharides [145]. Chitosan added to *T. chinensis* cells also conditioned a better response to different elicitation treatments [146]. Phenylethanoid glycoside accumulation in *Cistanche deserticola* cell suspension cultures dramatically improved after the addition of chitosan at optimal conditions [147]. Chitosan increased the production of trans-resveratrol 2.5-fold in *V. vinifera* cell suspension cultures [148] as well as enhancing the accumulation of anthraquinones, phenolics and flavonoids in *Morinda citrifolia* adventitious roots [149]. In a metabolic profiling after chitosan elicitation of *H. perforatum in vitro* roots, Brasili *et al.* observed a strong stimulation of secondary metabolism [150]. *Betula platyphylla* cell cultures treated with chitosan increased the accumulation of triterpenoids with antiviral, antibacterial, antitumor and anti-AIDS properties [151]. Adding chitosan improved artemisinin production in *A. annua* hairy roots [115] and flavonoid content in *Andrographis paniculata* cell suspension cultures [109]. Different concentrations of chitosan enhanced psoralen biosynthesis in cell cultures of *Psoralea corylifolia* and *Conium maculatum* [107,152] as well as the dicentrine content in *Stephania venosa* [85]. Finally, chipto-heptaosa, a chitosan derivative, markedly increased paclitaxel production in *T. cuspidata* cell cultures [153].

#### 4.3.2. Oligosaccharins

Fungus and plant cell walls are mainly composed of complex carbohydrates such as glucosamine polymers in the former and pectins, cellulose and hemicellulose in the latter. Some cell wall fragments, known as oligosaccharins, formed by the action of hydrolytic enzymes excreted by fungi and/or plant cells, have elicitor activity in plants. These compounds are short chains of sugar residues connected by glycosidic bonds, and at low concentrations they exert biological effects on plant tissues, initiating a plant’s response to attack by pathogens and insects. Oligosaccharins are also involved in several plant development processes [154,155].

The type and specificity of oligosaccharins’ effects vary according to their physicochemical characteristics and the plant species perceiving the signal. These compounds are recognized by different cell surface receptors, resulting in a direct stimulation of metabolic pathways and an increase in SAR. Some oligosaccharins have provided good model systems for studies on how plant cells recognize chemical signals (elicitors) and transduce them for activation of defense machinery. Glycoproteins have also been described as a source of oligosaccharins, which are artificially produced by the acid- or enzyme-catalyzed fragmentation of cell wall polysaccharides [156].

Yoshikawa *et al.* [157] studied receptors for glucan oligosaccharide elicitors by using radio-labelled elicitor-active polysaccharides, and demonstrated a single class of high affinity binding places in root membranes and other parts of soybean. Also, by use of labelling with l-[3*H*]arabinose and l-[3*H*]fucose *in vivo*, xyloglucan-derived oligosaccharides were found to accumulate extracellularly in spinach cell cultures [158].

*Phytophthora sojae* β-glucan acts as an active elicitor on various plant cells in the family Fabaceae, indicating the presence of similar perception systems among these plants [159]. In the case of tobacco cells, however, the hepta-β-glucoside did not act as an elicitor, while linear β-1,3-linked glucooligosaccharides (laminarioligosaccharides) were active elicitors [160].

Diosgenin production in *D. zingiberensis* cell cultures was enhanced by the action of oligosaccharides from the endophytic fungus *Fusarium oxysporum* [161]. Also, oligosaccharins (G-7 and G-8) obtained from *P. ginseng* had a positive effect on the production of red pigments in *Oamona panicuculatum* callus cultures [162].

#### 4.3.3. Other Cell Wall Fragments

Many other preparations or purified molecules derived from fungi and bacteria have been tested with positive results. Fungal exopolysaccharides extracted from *Fusarium oxysporium* mycelia increased diosgenin content 1.34-fold in *D. zingiberensis* cell suspensions [163]. In *V. vinifera* cell cultures pectin and alginate elicitation led to a 2.5- and 2.6-fold increase, respectively, in the production of stilbenes within 13 days of culture [148]. Pectin also improved anthraquinone, phenolic and flavonoid accumulation in adventitious root suspension cultures of *M. citrifolia* [149].

### 4.4. Other Elicitors

#### 4.4.1. Plant Regulator Peptides

Plant defense systems respond to herbivorous or pathogen attack by synthesizing hormones, reactive oxygen species (ROS), oligosaccharides and oligoprotein fragments. Plant cells also use small signaling peptides, composed of 5–75 amino acids, which can regulate or elicit plant metabolism, frequently cross-talking with hormone signaling cascades [164,165].

Small peptides have been shown to participate in plant resistance mechanisms. Systemin, the first signal peptide isolated from tomato leaves, regulates the synthesis of jasmonates and proteinase inhibitor proteins and the production of volatile organic compounds. In tobacco, two small hydroxyproline-rich glycopeptides have been described and included in the systemin family due to their defense signaling activities [166,167,168]. Other elicitor peptides, AtPep1 and ZmPep1, isolated from Arabidopsis and maize, respectively, promote defense gene expression by encoding defensin and endochitinase A, and regulate the production of benzoxazinoid, a defense secondary metabolite [169,170].

The plant endogenous elicitor, phytosulfokine-α (PSK-α), a 5-amino acid peptide, was obtained as an active compound from *Asparagus officinalis*, and induced mesophyll cultures with mitogenic activity [171]. This sulphated pentapeptide has also been identified in rice cell suspensions, increasing cellular growth and binding in plasma membranes, and is capable of inducing cell proliferation [172,173]. PSK-α was tested as an elicitor in *Atropa belladonna* hairy roots, affecting growth and tropane alkaloid production [174], and strongly elicited taxol production in *Taxus* cell suspension cultures with synergistic interaction with MeJa [175].

Screenings for new regulators of taxol biosynthesis, based on transcriptomic profile analysis of elicited *T. baccata* cell suspensions, have allowed the identification of a new master-regulator named Taximin. This cysteine-rich signaling peptide is encoded by a MeJa-responsive gene, whose expression increases in correlation with that of most taxol biosynthetic genes. Furthermore, synthetic Taximin exogenously added to a *Taxus* cell suspension was able to regulate taxane biosynthesis. The highly conserved nature of Taximin in plants probably explains the positive synergistic effect with MeJa on alkaloid biosynthesis in a Taximin-overexpressing *N. tabacum* hairy root line when MeJa was supplemented to the medium. These results suggest Taximin has a broad regulatory role in plant metabolism [176].

#### 4.4.2. Cyclodextrins

Cyclodextrins (CDs) are a family of macrocyclic oligosaccharides linked by α-1,4 glycosidic bonds to form a cone-shaped structure with a hydrophobic cavity. Interacting through non-covalent forces, this space allows CDs to form inclusion complexes with a variety of compounds, ranging from small molecules to proteins [177]. Widely studied since their discovery in 1981, the most common CDs include α-, β- and γ-CD, which are composed of 6, 7 and 8 glucose units, respectively. The low toxicity and immunogenicity of these molecules make their application in the pharmaceutical field highly attractive, and they have been used to form inclusion bodies with drugs [178].

CDs improve drug solubility, stability and absorption [179], masking odors and flavors [180], controlling the drug release profiles, alleviating local and systemic toxicity, and improving drug permeability through biological barriers [181].

In recent years, CDs have also attracted considerable attention as agents capable of inducing defense responses in plant cell cultures and therefore acting as true elicitors [182,183,184,185,186,187,188] (Table 2). In *Vitis vinifera*, CDs have been shown to trigger a signal transduction cascade that activates different families of transcription factors regulating the expression of genes related to the trans-resveratrol biosynthetic pathway. Moreover, as indicated previously, the chemical structure of these compounds allows them to form inclusion complexes with apolar compounds with low hydrosolubility, facilitating their excretion from cells and their isolation from the culture medium [189]. Thus, Belchi-Navarro *et al.* [190] have demonstrated that CDs stimulate the biosynthesis and extracellular accumulation of silymarin (a pharmacologically active flavolignan) in *Silybum marianum* cell cultures and Sabater-Jara *et al.* [55] in cell cultures of *T. media* showed that Taxol biosynthesis and excretion from the producer cells to the medium was clearly increased by the joint action of methyl jasmonate and CDs, reaching production levels 55 times higher than in non-elicited cultures.

**Table 1:** Effect of biotic elicitors on secondary metabolite production in plant *in vitro* cultures.

Elicitor	Culture System	Plant Species	Secondary Metabolites (SM)	Type of SM	Reference
MeJa	CS	*Linum album*	Podophyllotoxin; 6-methoxypodophyllotoxin	Polyphenols (Aryl tetralin-lignans)	[40]
	HR	*Linum tauricum*	4’-Dimethyl-6-methoxypodophyllotoxin; 6-methoxypodophyllotoxin	Polyphenols (Aryl tetralin-lignans)	[41]
	AR	*Panax ginseng*	Ginsenosides	Glycosylate triterpenes (Saponins)	[43,44,45,46,47,48,49]
	HR	*Panax ginseng*	Ginsenosides	Glycosylate triterpenes (Saponins)	[44,50]
	CS	*Panax ginseng*	Ginsenosides	Glycosylate triterpenes (Saponins)	[42,51]
	CS	*Taxus baccata Taxus media*	Paclitaxel and related taxanes	Diterpene alkaloids	[54,55,56,57]
	HR	*Hyoscyamos niger*	Scopolamine and hyoscyamine	Tropane alkaloids	[59]
	CS	*Vitis vinifera*	*trans*-Resveratrol and stilbenes	Phenylpropanoids	[60]
	CS	*Menthal x piperita*	Rosmarinic acid	Phenylpropanoyl	[61]
	CS	*Lavandula vera*	Rosmarinic acid	Phenylpropanoyl	[62]
	CS	*Thevetia peruviana*	Peruvioside	Cardiac glycoside	[63]
	HR	*Catharantus roseus*	Catharanthine	Tropanic alkaloid	[64]
	CS	*Centella asiatica*	Centellosides (Madecassoside, Asiaticoside)	Triterperne saponines	[65,66]
	CS	*Artemisia annua*	Artemisinin	Sesquiterpene lactone	[67]
SA	CS	*Taxus chinensis Taxus baccata*	Paclitaxel and related taxanes	Diterpene alkaloids	[72,73,74]
	CS	*Corylus avellana*	Paclitaxel	Diterpene alkaloids	[75]
	CS	*Linum album*	Podophyllotoxin	Polyphenols (Aryl tetralin-lignans)	[76]
	HR; AR	*Panax ginseng*	Ginsenosides	Glycosylate triterpenes (Saponins)	[77,78,79,80]
	CS	*Gingkgo biloba*	Bilobalide; ginkolide a, b	Sesquiterpenes	[81]
	CS; HR	*Hypericum* spp.	Cadensin G; Paxanthone	Xanthones	[82]
	CS	*Vitis vinifera*	Stilbenes	Phenylpropanoids	[83]
	AR	*Withania somnifera*	Withanolide a, b; withaferin a and whitanone	Withanolides	[84]
	CS	*Stephania venosa*	Dicentrine	Alkaloid	[85]
	CS	*Hypericum perforatum*	Hypericin; pseudohypericin	Naphtodianthrones	[86]
Bacteria extracts	HR	*Beta vulgaris*	Betalaine	Alkaloid	[101]
	HR	*Panax ginseng*	Ginsenosides	Glycosylate triterpenes (Saponins)	[80]
	CS	*Gingkgo biloba*	Bilobalide; ginkolides	Sesquiterpenes	[100]
	HR	*Salvia miltiorrihiza*	Tanshinone	Diterpene	[102]
	HR; CS	*Panax ginseng*	Ginsenosides	Glycosylate triterpenes (Saponins)	[28,80]
	CS	*Linun album*	Podophyllotoxin	Polyphenols (Aryl tetralin-lignans)	[29]
	CS	*Silybum marianum*	Silymarin	Flavonolignans	[30]
	HR	*Artemisia annua*	Artemisinin	Sesquiterpene lactone	[115]
	HR	*Fagopyrum tataricum*	Rutin; quercetin	Flavonoids	[104]
	HR	*Salvia miltiorrihiza*	Rosmarinic acid	Phenylpropanoids	[116]
	CS	*Cayratia trifolia*	Stilbene	Phenylpropanoids	[114]
Fungal extract	CS	*Hypericum perforatum*	Hypericin; pseudohypericin	Naphtodianthrones	[105]
	CS	*Coleus forskohlii*	Forskolin	Diterpene	[106]
	CS	*Psoralea corylifolia*	Psoralen	furocoumarins	[107]
	CS	*Euphorbia pekinensis*	Euphol	Terpenoids	[108]
	CS	*Andrographis paniculata*	Flavonoids	Flavonoids	[109]
	CS	*Linun album*	Podophyllotoxin	Aryl tetralin-lignans	[112,113]
	HR	*Linun album*	Podophyllotoxin; 6-methoxypodophyllotoxin	Aryl tetralin-lignans	[122,124]
	CS	*Taxus* spp.	Paclitaxel and related taxanes	Diterpene alkaloids	[117,118,119,123]
	CS	*Panax ginseng*	Ginsenosides	Glycosylate triterpenes (Saponins)	[120]
	HR	*Panax ginseng*	Ginsenosides	Glycosylate triterpenes (Saponins)	[121]
Coro	CS	*Corylus avellana*	Taxanes	Diterpene alkaloids	[136]
	CS	*Taxus media Taxus globosa*	Paclitaxel and related taxanes	Diterpene alkaloids	[131,138]
	CS	*Linun nodiflorum*	6-Methoxypodophyllotoxin	Aryl tetralin-lignans	[140]
	CS	*Eschoscholzia californica*	Benzo[c]phenanthridine	Alkaloid	[133]
	CS	*Glicine max*	Glyceollins	Isoflavonoid	[134]
Chitosan and chitin	CS	*Linun album*	Podophyllotoxin	Aryl tetralin-lignans	[112,142]
	CS	*Taxus chinensis Taxus canadiensis*	Paclitaxel	Diterpene alkaloids	[143,144,145]
	CS	*Cistanche deserticola*	Phenylethanoid glycoside	Phenylethanoid glycoside	[147]
	CS	*Vitis vinifera*	Stilbenes; trans-resveratrol	Phenylpropanoids	[148]
	AR	*Morinda citriflora*	Total anthraquinones, phenolics and flavonoids	Anthraquinones, phenolics and flavonoids	[149]
	CS	*Betula platyphilla*	Total triterpenoids	Triterpenoids	[151]
	HR	*Artemisia annua*	Artemisinin	Sesquiterpene lactone	[115]
	CS	*Andrographis paniculata*	Flavonoids	Flavonoids	[109]
	CS	*Psoralea corylifolia Conium maculatum*	Psoralen	Furocoumarins	[107,152]
	CS	*Stephania venosa*	Dichentrine	Alkaloid	[85]
Other cell Wall fragments	CS	*Dioscorea zingiberensis*	Diosgenin	Saponin	[163]
	CS	*Vitis vinifera*	Stilbenes; trans-resveratrol	Phenylpropanoids	[148]
	AR	*Morinda citriflora*	Total anthraquinones, phenolics and flavonoids	Anthraquinones, phenolics and flavonoids	[149]
	CS	*Taxus cuspidata*	Paclitaxel	Diterpene alkaloids	[153]

MeJa: Methyl jasmonate; SA: Salicylic acid; Coro: Coronatine; CS: Cell suspensions; HR: Hairy roots; AR: adventitious roots.

Recently, a new kind of elicitor has attracted attention: insect- and plant-derived compounds found in insect oral secretions such as voliticin, inceptins, caeliferins. These compounds act as elicitors by activating JA responses as well as the production of secondary metabolites, mainly volatiles. Despite their potential importance, few studies have applied these new elicitors to plant cell cultures to date.

**Table 2:** Effect of cyclodextrins on secondary metabolite production in plant *in vitro* cultures.

Culture System	Plant Species	Secondary Metabolite (SM)	Type of SM	Reference
CS, HR	*Taxus globosa*	Taxanes	Diterpene alkaloid	[138]
	*Morinda citrifolia* and *Rubia tinctorum*	Anthraquinones	Phenolic compounds	[182]
	*Catharanthus roseus*	Vindoline, catharanthine and ajmalicine	Terpenoid indole alkaloids	[183]
	*Taxus media*	Taxanes	Diterpene alkaloid	[55]
	*Catharanthus roseus*	Ajmalicine	Monterpenoid indole alkaloid	[184]
	*Vitis vinifera*	Trans-resveratrol	Stilbenes	[60,185,186,187]
	*Scutellaria lateriflora*	Verbascoside, the flavones: wogonin, baicalein, scutellarein and their respective glucuronides	Phenolic compounds	[188]

CS: Cell suspensions; HR: Hairy root cultures.

## 5. Conclusions and Perspectives

An understanding of how plant tissues and their specific secondary metabolic pathways respond to abiotic and biotic elicitors, applied individually or in combination, is essential for designing strategies for an enhanced biotechnological production of bioactive compounds. One of the main obstacles for PSM production systems is the limited knowledge of highly complex biosynthetic pathways and their controlling enzymes and genes, as well as the lack of elucidation of the relevant transcription factors and master regulators.

New insights into incompletely defined biosynthetic routes are being provided by state-of-the-art “omics” technologies. Proteomics and metabolomics can shed light on the elicitation of plant secondary compounds and their relation with primary metabolism. The continuing study of transcriptomic profiles and how genes are differentially expressed after elicitation, along with the production patterns of target secondary compounds, will uncover limiting metabolic steps and identify potential targets for engineering. “Omics” technologies can also modify these secondary pathways by the overexpression or inhibition of regulatory genes encoding transcription factors or master regulators. With greater metabolic knowledge, the accurate application of elicitor-driven effects at selected culture time points may be a successful strategy to obtain tailored highly productive cell cultures. Nevertheless, it has been shown that even cell cultures engineered to overexpress key genes of a selected biosynthetic pathway still need elicitation to achieve high production levels. Therefore, the selection of the most suitable elicitor for a plant cell culture will remain crucial.

Current progress in the application of synthetic biology technologies has allowed the production of high added value secondary compounds in heterologous systems to become almost a reality. The definitive breakthrough in a cost-effective and sustainable commercial production of PSM will depend on a more in-depth knowledge of the metabolic response to elicitation in plant cells, as well as a thorough understanding of the mechanisms responsible for these effects.
